# Multiomics technologies for comprehensive tumor microenvironment analysis in triple-negative breast cancer under neoadjuvant chemotherapy

**DOI:** 10.3389/fonc.2023.1131259

**Published:** 2023-05-22

**Authors:** Gang Wang, Yao Yao, Huanhuan Huang, Jun Zhou, Chao Ni

**Affiliations:** ^1^Department of Surgical Oncology, The First Affiliated Hospital of Bengbu Medical College, Bengbu, Anhui, China; ^2^Department of Breast Surgery, Second Affiliated Hospital, Zhejiang University, Hangzhou, Zhejiang, China; ^3^Key Laboratory of Tumor Microenvironment and Immune Therapy of Zhejiang Province, Second Affiliated Hospital, Zhejiang University, Hangzhou, Zhejiang, China; ^4^Cancer Center, Zhejiang University, Hangzhou, China; ^5^Department of Breast Surgery, Affiliated Hangzhou First People’s Hospital, Zhejiang University, Hangzhou, Zhejiang, China

**Keywords:** neoadjuvant chemotherapy, triple-negative breast cancer, tumor microenvironment, multi-omics, single-cell analysis

## Abstract

Triple-negative breast cancer (TNBC) is one of the most aggressive breast cancer subtypes and is characterized by abundant infiltrating immune cells within the microenvironment. As standard care, chemotherapy remains the fundamental neoadjuvant treatment in TNBC, and there is increasing evidence that supplementation with immune checkpoint inhibitors may potentiate the therapeutic efficiency of neoadjuvant chemotherapy (NAC). However, 20-60% of TNBC patients still have residual tumor burden after NAC and require additional chemotherapy; therefore, it is critical to understand the dynamic change in the tumor microenvironment (TME) during treatment to help improve the rate of complete pathological response and long-term prognosis. Traditional methods, including immunohistochemistry, bulk tumor sequencing, and flow cytometry, have been applied to elucidate the TME of breast cancer, but the low resolution and throughput may overlook key information. With the development of diverse high-throughput technologies, recent reports have provided new insights into TME alterations during NAC in four fields, including tissue imaging, cytometry, next-generation sequencing, and spatial omics. In this review, we discuss the traditional methods and the latest advances in high-throughput techniques to decipher the TME of TNBC and the prospect of translating these techniques to clinical practice.

## Introduction

**Figure 1:**
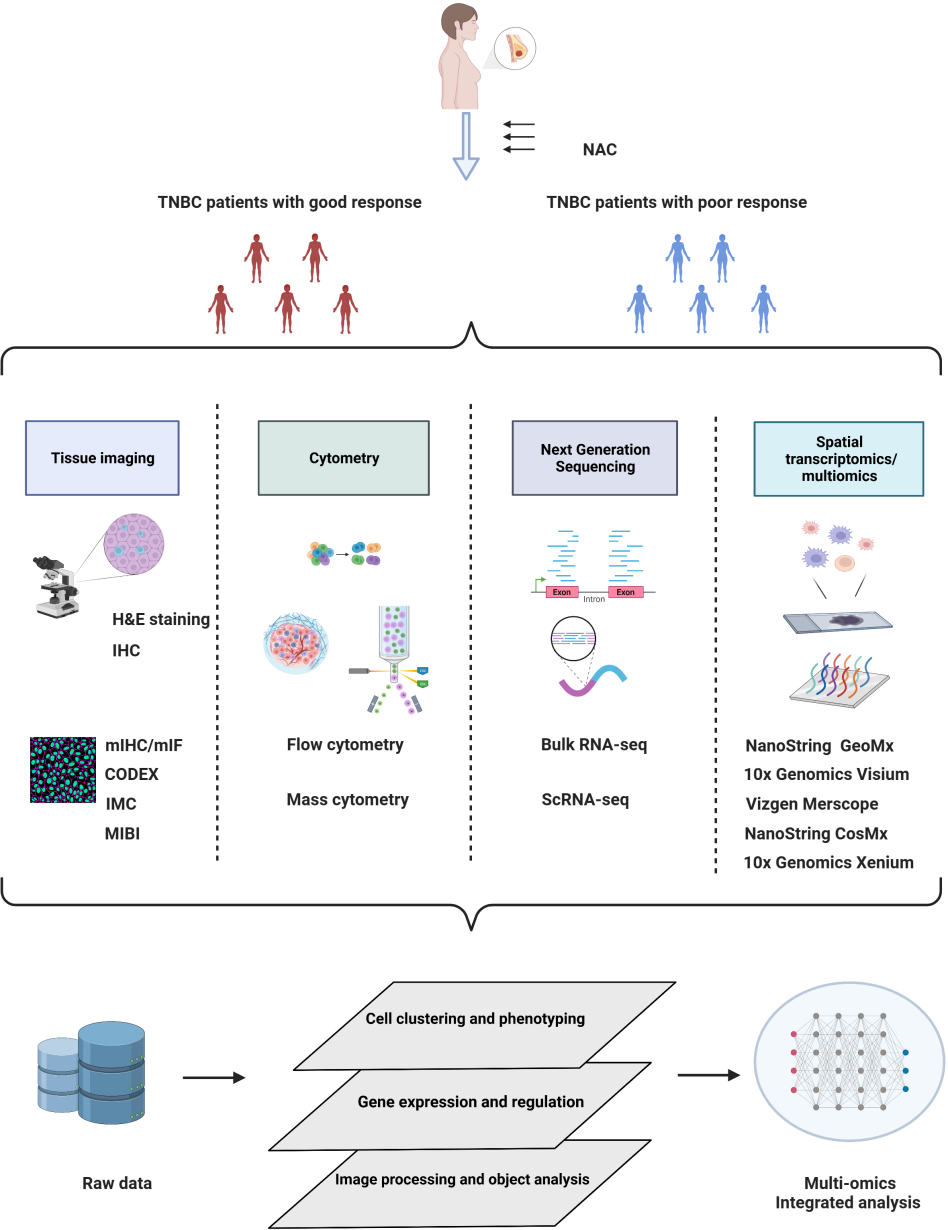
Schematic overview of multiomics approaches in TNBC studies under neoadjuvant chemotherapy. Elucidation of the TME by multiomics tumor profiling may enhance the development of novel biomarkers and therapeutic targets for NAC treatment. Created with BioRender.com.

Triple-negative breast cancer (TNBC), defined by the absence of estrogen receptor (ER), progesterone receptor (PR), and human epidermal growth factor receptor 2 (HER2), accounts for approximately 25-30% of all breast cancers and exhibits poor prognosis and strong invasiveness (1) As the standard treatment strategy for early-stage TNBC, neoadjuvant chemotherapy (NAC) not only provides opportunities for breast conservation and sparing axillary lymph node dissection but also identifies patients with residual disease at high risk of relapse (2). Although approximately 30% of TNBC patients who receive NAC achieve a complete pathological response (pCR) (3), the remaining patients with residual disease require supplemental adjuvant chemotherapy or targeted regimens. Given that tumor-infiltrating lymphocytes (TILs) have been found to be more extensive in TNBC than in other breast cancer subtypes, randomized clinical trials have been conducted to investigate the strategy of combining immune checkpoint inhibitors (such as anti-PD-1 and anti-PD-L1) with traditional chemotherapy in NAC. However, the discrepancy in the results among relevant clinical studies suggests that the dynamic changes in the tumor immune microenvironment (TME) need to be elucidated under different treatments (4–7). Traditional methods, including immunohistochemistry, bulk tumor sequencing, and flow cytometry, may not comprehensively elucidate the TME of breast cancer, and the function of some subsets of cells present in relatively low proportions may also be underestimated (8–10). In addition, the dynamic TME landscape of pre- and post-NAC breast cancer is still poorly studied with these methods (11–13). With the development of diverse high-throughput technologies and bioinformatics analysis methods, recent reports have opened a new horizon of NAC-induced alterations in tissue imaging, cytometry, next-generation sequencing, and spatial omics at the single-cell level in breast cancer (**Figure 1**). In this review, we discuss the latest updates on immune infiltrate alterations in the multiomics layer and present a structured review of traditional pathological and emerging bioinformatics approaches in terms of the TME in TNBC patients receiving neoadjuvant treatments, which may help to improve the understanding of how NAC remodels the TME in TNBC.

## Tissue imaging

### Hematoxylin and eosin staining

To date, morphological identification of TILs in breast cancer with hematoxylin and eosin (H&E) staining remains the most traditional and widely applied method. In 1990, Crescitelli et al. first reported that lymphocyte infiltrates were an independent critical predictor of breast cancer, particularly in rapidly proliferating, axillary lymph node-negative tumors, compared to slowly proliferating or axillary lymph node-positive tumors (14, 15). In a retrospective study that collected 1058 samples from two NAC clinical trials with H&E-stained sections, the presence of tumor-associated lymphocytes in breast cancer was first reported as an independent predictor of response to anthracycline/taxane-based neoadjuvant chemotherapy (15). In addition, intraepithelial lymphocytes inside tumor cell nests or in direct contact with tumor cells were defined as intratumoral TILs, whereas lymphocytes in the tumor stroma without direct contact with tumor cells were defined as stromal TILs; both types were confirmed to have predictive value for pCR. With evidence accumulating for a strong correlation between TILs and NAC response in breast cancer, the specialist consensus on the standardized method to evaluate TILs based on H&E-stained tissue sections was published by the International TILs Working Group in 2014 (16). In this consensus, the area selected for TIL evaluation and assessment methodology was described in detail; however, the interobserver variability remains substantial in the assessment of stromal TILs (17) or intratumoral TILs (18). To overcome the heterogeneity among pathologists and among guidelines, TIL evaluation assisted by machine-learning technology for TIL scoring is under development (19–21), mainly aiming to improve analytical standardization and reproducibility. In two interlaboratory comparison trials using identical samples, the intraclass correlation coefficient (ICC) for TIL evaluation among 32 pathologists in Ring Study 1 was 0.7, whereas in Ring Study 2, a software-guided image evaluation system enhanced the ICC to 0.89 (22). Computer-assisted TIL assessment holds promise for complementarity to the present pathological examination of breast tumors. Manual TIL counting fails to count all cells and measure the precise stromal area in the entire sample. Recently, Eva et al. presented a fully automated digital image analysis pipeline and demonstrated that their H&E-based pipeline provides a quantitative and interpretable score comparable with manually derived TIL status (19).

Although computational assistance technology has greatly improved the efficiency and reproducibility of TIL counting, the analyses are still based on the gross morphology of the lymphocyte entirety on H&E slides. Quantitative assessment of polymorphonuclear leukocytes, granulocytes, and other mononuclear cells, such as dendritic cells and macrophages, is typically not mentioned (16). In addition, increasing evidence suggests that the changes in the proportion and phenotype of various immune subpopulations are inconsistent during NAC (23, 24). Therefore, a more thorough assessment is required to decipher the relative proportion of specific immune subpopulations and spatial structural features, including tertiary lymphoid structures (25).

### Conventional immunohistochemistry

Conventional immunohistochemistry (IHC) is one of the most common methods for investigating the immune content of a tissue by detecting characteristic markers using specific antibodies. Compared to H&E staining, IHC allows labeling of different immune cell compositions to determine their association with treatment response or tumor prognosis, including TNBC. In a retrospective study based on double-staining immunohistochemistry, Miyashita et al. first reported that high CD8+ TILs and a high CD8/FOXP3 ratio in residual tumors following NAC exhibit strong prognostic significance in TNBC patients (26). IHC for PD-L1 has been performed on paraffin-embedded tumor samples from core needle biopsies from 94 breast cancer patients before NAC, identifying PD-L1 expression as a predictor of pCR and a prognostic factor of disease-free survival (DFS) in breast cancer patients (27). In addition, Gomez-Macias et al. assessed the TIL profile in TNBC before NAC by IHC on core needle biopsies, and they identified a more accurate association of the high expression levels of the CD3, CD4, CD8, CD45, and CD20 genes with partial and complete pathological responses to NAC in patients with TNBC (28).

With the advantages of rapid and feasible processing and low cost, IHC is still widely used in studies despite a variety of emerging technologies that have been developed. However, traditional IHC is a semiquantitative and observer-dependent technique. In terms of the IHC assessment of PD-L1 expression, the definition of a PD-L1+ sample has not been completely established to date. Some researchers solely detect PD-L1 on tumor cells or immune cells, whereas others evaluate PD-L1 expression regardless of cell type (29), which may affect the consistency of PD-L1 evaluation among different research platforms. In addition, similar to TIL counting based on H&E staining, the inevitably high interobserver variability of biomarker assessment by IHC is another impediment. In a study assessing interobserver variation of PD-L1 IHC (SP142 antibody) in breast cancer, 38% of cases did not reach total agreement among all eight pathologists (30). PD-L1 expression in immune cells (0.172-0.229) evaluated by pathologists has also shown poor consistency (ICC, 0.832-0.882) (31). In addition, it has been reported that multiple variables, such as tissue processing, antibodies, staining design, and scoring systems, may also affect IHC results (32, 33). For the analysis of small and precious tumor samples, such as core needle biopsy or unconventional proteins, the application of IHC may be restricted due to the uncertainty of antibody concentration. However, a major limitation of conventional IHC is that it only identifies a single or at most two markers per tissue section, while some cell subsets require multiple markers to be defined. Therefore, conventional IHC fails to meet the needs of researchers, and the multiplex fluorescent IHC approach has been utilized as a solution.

### Multiplex immunohistochemistry/immunofluorescence

Multiplex immunohistochemistry/immunofluorescence (mIHC/IF) offers an upgraded strategy over standard chromogenic IHC due to multiplex staining and standardized quantitative analysis (34). Using a multispectral IF (mIF) platform, the quantification of six to eight markers in a single FFEP tissue section can be achieved (35). The mIF image analyzed by inForm image software correlates the spectral signals to biomarkers and histomorphology information, achieving cell type identification and standardization of quantitative analysis. A quantitative immunofluorescence (QIF) assay was first developed by Brown et al. in 2014 for measuring CD3, CD8, and CD20 expression on a single slide, which confirmed the predictive value of different lymphocyte subsets in breast cancer following NAC (36). Of note, mIHC/IF depicts the spatial biology of the TME and achieves both quantification and localization of immune marker expression. Mani et al. used PerkinElmer Vectra multiplexed QIF to analyze the location and heterogeneity of TILs by quantifying cytokeratin, CD3, CD8, and CD20 expression in tissue sections from different breast cancer subtypes (37). Furthermore, mIF is available to assess treatment-related effects on specific cell lineages in both the stromal and tumor compartments. Applying Akoya Bioscience OPAL 7-plex mIHC to the first cycle of NACT in the WSG-ADAPT-TN trial (n=66 TNBC patients), the increase in PD-1-+ CD4 and PD-1+ CD8 infiltration in both the tumor and in the stroma were significantly correlated with higher pCR (38). In another neoadjuvant PAMELA phase II trial, 231 regions of interest (ROIs) from 129 HER2+ breast cancer patients were stained with six markers, including cytokeratin, Ki67, and four immune-related T-cell lineage markers (CD3, CD4, CD8, and Foxp3). Using the Discovery Ultra Autostainer (Ventana Medical Systems, Tucson AZ), the researchers found that the distance between CD3+/CD8+ T cells and cancer cells was shortened under anti-HER2-based neoadjuvant treatment, indicating the strong predictive value of the spatial relationships of specific immune cell subsets and tumor cells (39).

The main advantage of mIHC/IF is the simultaneous detection of multiple markers on a single tissue section. Detecting various markers on a single section is crucial due to the low availability of NAC tissue samples. In addition, the objectivity, accuracy, and sensitivity of QIF have been confirmed in comparison with IHC (40). Previous research has suggested that PD-L1 expression assessed by IHC is not sufficiently sensitive to predict the response to anti-PD-1/PD-L1 treatment (41, 42). By reviewing tumor specimens from 8135 patients with over 10 solid tumor types, Taube et al. found that mIHC/IF showed a higher accuracy in predicting the clinical response to anti-PD-1/PD-L1 therapy than PD-L1 IHC, and the former involved the analysis of PD-1 to PD-L1 proximity and intratumoral/peritumoral CD8+ cell density (43). Additionally, mIF combined with hierarchical linear modeling allows for a more precise estimation of TILs and PD-L1 expression increases by lessening the impact of intratumoral heterogeneity on cell counting (44).

Although mIHC/IF has achieved great advances, applying the technology in routine clinical practices still faces obstacles due to the complex staining procedure, longer turnaround time than traditional IHC, and requirements of antibody optimization, panel optimization, experienced pathologists, and sophisticated software. Therefore, several commercial kits, such as TSA-Opal automation IHC kits and commercial multispectral mIF platforms from Akoya Biosciences, have been developed to evaluate immuno-oncology targets in FFPE tumor tissue, making the process more feasible, efficient, and reproducible. Moreover, a reported mIHC/IF staining protocol applied to the commonly used clinical diagnostic autostainer, namely, the Leica Bond Max, enhances the translation to clinical routine (45). A previous study using an Opal 7-color automated IHC detection kit on a Leica Bond Rx autostainer has reported that it requires approximately 12-13 h to complete a six-plex assay on 30 slides concurrently, and high reproducibility in measuring immune cell density and PD-L1 coexpression has been demonstrated among six laboratories (46). Similarly, a commercial mIF assay, known as InSituPlex, can be performed on an existing fluorescence microscope, simplifying the staining procedure (47). For example, Rimm et al. retrospectively analyzed pretreatment core needle biopsies obtained from 69 TNBC patients with an Ultivue DNA-based Ultimapper kit. The authors suggested that, in patients who achieved pCR under NAC, PD-L1 expression was significantly higher in tumor cells, CD68+ cells, and stroma than in nonpCR patients. In contrast, the number of total CD68+ cells in the tumor or stromal compartments was similar between pCR and nonpCR cases (48).

### Spatial proteomics

Traditional imaging technology fails to depict the TME in detail due to low plex. For an in-depth understanding of the TME, it is crucial to capture the spatial proteome, including subcellular protein localization and the dynamic relationship between subcellular protein localization and protein function. Accumulating spatial proteomics technologies, including GeoMx Digital Spatial Profiler (DSP) and cyclic immunofluorescence-based CODEX and Multi-Omy as well as mass spectrometry (MS)-based imaging mass spectrometry (IMS) and multiplex ion-beam imaging (MIBI), are available for deconvoluting the spatial constitution of the TME at the single-cell level or close to the single-cell level, and the distinctive features of these technologies have been comprehensively discussed previously (49). Here, we mainly review these novel findings in breast cancer, particularly in TNBC, based on these high-throughput multiplex staining methods. A representative spatial proteomics technology based on fluorescence imaging is the codetection by indexing (CODEX) single-cell proteomics analysis platform developed by Akoya (50). With the application of CODEX to quantify immune infiltration inside quiescent cancer cells (QCCs) in a TNBC mouse model, Baldominos et al. confirmed markedly reduced immune infiltration within the QCC niche, and they also identified DCs with lower MHCII and exhausted T cells within the QCC niche (51). Imaging mass cytometry (IMC) and multiplexed ion beam imaging (MIBI) are typical mass spectrometry-based spatial proteomics techniques. IMC allows simultaneous multiplexed imaging of up to 40 proteins with subcellular spatial resolution (52, 53). Simultaneous multiplexed detection of mRNA, proteins, and protein phosphorylation through imaging mass cytometry has been achieved in a cohort of 70 breast cancer samples, revealing the significant spatial interactions among CXCL10^high^ cells (macrophages, fibroblasts, T cells, and epithelial cells) with each other (54). Another mass cytometry imaging approach is MIBI (55), which uses the secondary ionization mass spectrometry principle, whereas IMC utilizes laser ablation. Keren et al. performed the first study capturing features and the spatial orientation of immune cells within the TME *via* MIBI from 41 TNBC patients (56); they divided tumors into cold, mixed, and compartmentalized subtypes by assessing the spatial proximity of cell types, and highly ordered structures with PD-L1 and IDO along the tumor-immune border served as a dominant feature of tumor compartmentalization and were associated with favorable survival.

## Cytometry

### Flow cytometry

Compared to H&E staining or IHC, flow cytometry (FCM) is a relatively high-throughput technology extensively used in studies on cancer immunity that enables the rapid quantification of multiple parameters of immune cell populations or particles. Based on single-cell suspensions, including dissociated solid tissue and various body fluids, FCM can measure cell size, granularity, and the expression level of cell surface and intracellular molecules (57, 58). Compared to IHC, the advantage of FCM is that it incorporates quantitative and functional evaluation of tissues and circulating immune cell repertoires, whereas IHC is limited to the tissue level. However, tissue biopsy during NAC is challenging, and circulating immune cells have been extensively evaluated during NAC. Previous studies have indicated that these circulating cells may also be informative and capable of serving as promising biomarkers to predict treatment response (59–61). A study quantifying myeloid-derived suppressor cells (MDSCs) and Tregs by flow cytometry in blood samples from 34 pre-NAC TNBC patients has revealed a negative correlation between early MDSC (eMDSC) levels and NAC response (61). In contrast, the peripheral blood level of granulocytic MDSCs (G-MDSCs) is also increased during chemotherapy with doxorubicin and cyclophosphamide but has no relation to pCR (10). By detecting the expression of surface receptors on CD8+ T cells before and four months after NAC, it has been found that an increase in P2X7 expression and a decrease in A2A receptor expression indicate a better response (62). In addition, FCM is also capable of detecting the expression of intracellular proteins, which allows researchers to determine the functional state of immune cells (57). For example, granzyme B/perforin levels in circulating NK cells have been reported to be reduced during NAC, which is the most significant decrease that appears in advanced breast cancer patients with poor response (63). In another study that collected a series of blood samples from 56 TNBC patients under NAC and extensively evaluated circulating immune cells, B cells, NK cells, and CD4+ T lymphocytes were greatly decreased, whereas the frequency of CD8+ T cells was less affected (64). In addition, functional property analysis has revealed that the expression of the Tim3 exhaustion marker is markedly upregulated in CD8+ T cells, whereas cytotoxic molecules, including perforin and CD3ζ, are lost in NK cells (64).

FCM is a powerful technique for analyzing a single cell; the latest FCM system allows the detection of up to 40 fluorescent parameters. However, the accuracy of the results may be easily affected by the spectral overlap of the fluorescent dyes, especially for cell subsets with low proportions (65, 66). Moreover, the lack of fluorescently labeled antibodies used for certain proteins also limits the application of this method. By analysis of the phenotype or function of cell subpopulations in tissues using flow cytometry, the morphological parameters of the cells are altered when the tissue is enzymatically treated or mechanically dissociated, and the information regarding the spatial location of cells within the whole tissue is lost (67) Therefore, a variety of techniques need to be combined to study the characteristics of the TME at the cellular and spatial levels.

### Automated FCM analysis and CyTOF

With significant developments in both lasers and fluorophore reagents, the number of parameters of FCM has been extended from 17 (65) to 40 (68). However, the analysis of traditional FCM data is still dependent on manual gating strategies, which may create interpersonal bias, particularly for multicolor labeling strategies (69). Thus, the improved multiparameter capabilities of FCM require computational flow cytometry to visualize high-dimensional cytometry data to improve accuracy and reproducibility (70). A computational pipeline known as FlowGM, with automated identification of 24 cell types, has been demonstrated to better discriminate monocyte and dendritic cell subpopulations than the traditional gating strategy (71). Other automated cell identification tools, including PhenoGraph (72), SPADE3 (73), FlowSOM (74), SWIFT (75), t-SNE (76), and UMAP (77), distinguish cell populations from cytometry data in both unsupervised and supervised manners (78, 79).

An emerging fusion technology known as mass cytometry or cytometry by time-of-flight (CyTOF) avoids spectral overlap by replacing the fluorophores of probes with isotopically purified metals, which are then detected and quantified by inductively coupled plasma time-of-flight mass spectrometry (ICP-TOF-MS) (80, 81). With the capacity to monitor 34 parameters simultaneously in individual cells, CyTOF enhances the sensitivity of identifying rare cell subpopulations (81, 82).

However, the throughput of mass spectrometry is required to collect millions of cells from an individual sample (80), which is not practical for small tumor and needle biopsy specimens. Thus, to bypass the scarcity of some immune cells, using mass cytometry in the field of peripheral blood samples from breast cancer patients is a promising strategy (83). In a phase 1/2 clinical trial of the nitric oxide synthase inhibitor, L-NMMA, and taxane in TNBC, by analyzing PBMCs using CyTOF (Fluidigm) from two responders and two nonresponders, CD8 effector memory T cells and CD4+ T cells have been found to be increased in responders, whereas nonresponders show a more classical monocyte immunotype (84). Because CyTOF is applied only to cell suspensions, spatial information and cell−cell interactions are lost (85, 86). Preservation of spatial information requires the combination of IHC and CyTOF. With the application of immunohistochemistry, immunocytochemistry, and CyTOF (Fluidigm) in breast cancer FFPE, Giesen et al. achieved higher multiplexed applications at subcellular resolution to depict substantial tumor microenvironment heterogeneity (53).

## Next generation sequencing

### Bulk tumor RNA sequencing

Deep insight into NAC-induced alterations at the transcriptomic level has been provided through unbiased RNA-seq of paired biopsy specimens collected at a series of time points (**Table 1**). Bulk tumor RNA sequencing is one of the most valuable and widely used methods for analyzing differential gene expression among various samples (80). Potential cancer biomarkers are continuously being discovered with RNA sequencing (96). For example, by analyzing the expression of 750 immune-related genes from 60 paired pre- and posttreatment breast cancer samples, a set of immune parameters adversely correlated with pCR has been identified, suggesting that regimens targeting mast cell metagenes as well as VEGFB, IL-6 antagonists, and anti-VEGF antibodies may enhance the sensitivity of the tumor to NAC (88). The strongly negative prognostic gene, CDH1, and the positively prognostic gene, CD70, have been identified in the TNBC cohort by analysis of immune gene expression during NAC with the Pan-Cancer Immunology 770 genes panel(NanoString) (89). In post-NAT residual tumors from 62 patients treated with immune gene expression during NAC and anti-EGFR antibodies, 784 genes have been processed( NanoString nCounter), identifying SOX2 and CXCR4 as potential recurrence predictors (91). In addition, by evaluating Affymetrix microarray transcriptomic data from more than 2000 TNBC patients, immune signatures, including IDO1, CXCL9, CXCL10, HLA-DRA, HLA-E, STAT1, and GZMB, have been found to be associated with a favorable prognosis in TNBC patients (97). However, a prospective study with sufficient cohorts is required to validate and confirm the utility of biomarkers.

**Table 1:** NAC studies with transcriptome analysis (characteristics, prognosis, and prediction).

Year	Treatment		BC type	Sampling time	Sampling type	Patient number	Assay	Main findings		Clinical cohort
2018 (87)	Epirubicin docetaxel		TNBC	Pretreatment and during treatment	Tumor tissue	20	scRNAseq	Chemoresistance-related gene signatures: EMT, CDH1 targets, AKT1 signaling, hypoxia, angiogenesis, and ECM degradation		NCT00957125
2019 (88)	Bevacizumab nab-paclitaxel doxorubicin cyclophosphamide		HER2-negative, locally advanced, or ICB	Pre-and posttreatment	Tumor tissue	60	NanoString PanCancer IO360™	Positive prognostic biomarkers: CCL21, CCL19, and cytotoxic T cell	Negative prognostic biomarkers: CXCL1, CXCL3, CXCL2, CCL20, and IL6	NCT00856492
2020 (23)	AC, T, H		BC	Pretreatment, during, and post- treatment	Tumor tissue	146	Illumina HiSeq2500	Immune stimulatory response on treatment rather than baseline is more predictive.		NCT02591966
2020 (89)	Neoadjuvant therapy		BC	RD	Tumor tissue and peripheral blood	83	NanoString PanCancer Immunology panel Isoplexis TCR sequencing ScRNAseq	Positive prognostic biomarkers: cytotoxic effector genes	Negative prognostic biomarkers: eight-gene signature (PDCD1 + NKG7 + LAG3+ GZMH + GZMB + GNLY + FGFBP2 – HLA-G) model in peripheral blood	DART EA1311 PERU
2021 (90)	nab-paclitaxel pembrolizumab		TNBC	Pretreatment and during treatment	Tumor tissue	2	ScRNAseq	Positive predictors: IFN+ and GZMB+ CD8+ T cells as well as TRM cells	Negative predictors: myeloid cells and lack of PD-1^high^ T cells	NCT02752685
2021 (91)	panitumumab trial: panitumumab, FEC, T cetuximab trial:cetuximab, and T		TNBC	Pre-and posttreatment	Tumor tissue	62	NanoString	Positive predictor: HLA class I or II	Negative predictors related to metastasis: cell cycle-related genes as well as SOX2 and CXCR4	NCT00933517 NCT00600249
2021 (92)	Half paclitaxel monotherapy Half atezolizumab plus paclitaxel		TNBC	Pretreatment and during treatment	Tumor tissue and peripheral blood	22	scRNAseq scATACseq	Positive predictors: CD8-CXCL13 T cells CD4-CXCL13		NA
2021 (93)	NAC		TNBC	Pre- and posttreatment	Tumor tissue	8	scRNAseq	Positive predictors: CD19, CD8A, CD4, CD52, CD2, CD53, CD59, CD47, CD74, and CXCL9		NA
2021 (94)	NAC		TNBC	Pretreatment and during treatment	Tumor tissue	6	Single-cell lncRNA transcriptome		Negative predictors: MALAT1, USP3-AS1, and LINC-PINT	NA
2022 (95)	NAC		BC	Pretreatment and during treatment	Peripheral blood	94	TCRβ sequencing	Positive predictors: Vβ20.1 and Vβ30		NA

EMT, epithelial-mesenchymal transition; IBC, inflammatory breast cancer; FEC, 5-fluorouracil+epirubicin+cyclophosphamide; T, Taxol (docetaxel); H, Herceptin (trastuzumab); RD, residual disease; TRM T cells, tissue resident memory T cells; lncRNA, long noncoding RNA; NA, not available.

By utilizing approaches based on marker gene expression values (including MCP-counter and xCell) or deconvolution algorithms (including TIMER, CIBERSORT, quanTIseq, and EPIC), it is practicable to reflect the content and subpopulations of tumor-infiltrating immune cells (98–100). Among these methods, EPIC and quanTIseq are the only methods providing an absolute score; MCP-counter and CIBERSORT are recommended in some cases requiring relative scores, and xCell is suggested when focused on a specific cell type (101, 102). For example, regulatory CD4+ T cell (Treg) abundance has been calculated by the xCell algorithm from bulk tumor gene expression data of 5177 breast cancer patients from five independent cohorts, showing the potential of Treg abundance as a biomarker for predicting the response to NAC in TNBC (103). Another RNA-seq profiling analysis that included 110 pairs of serial tumor biopsies collected before NAC, after the first treatment cycle, and at the time of surgery used CIBERSORT to reveal the relative fractions of ten immune cell types among tumor-infiltrating leukocytes, showing an increased abundance of CD4+ and CD8+ T cells induced by the first cycle of NAC (23).

In addition to bulk tumor sequencing, T cell receptor (TCR) sequencing has also been applied to assess the changes in T cell diversity and function induced by NAC in TNBC. TCR is a vital T cell response mediator that acts through antigen recognition (104). TCR sequencing identifies T cell clone TCRs and assesses their response to tumor antigens, offering an accurate perspective of T cells (105). In a cohort of eight formalin-fixed paraffin-embedded (FFPE) samples from TNBC, T cell receptor (TCR) β chain sequencing and clonality quantification have demonstrated a downward trend in T cell clonality but recruitment of novel clones during the NAC period (89). Another TCR sequencing study of peripheral blood samples from 94 breast cancer patients has shown that the diversity of the circulating TCRβ repertoire gradually decreases during NAC and is positively correlated with a better response (95). This effect is consistent with several findings that newly recruited tumor-specific T cells from outside the tumor play a crucial role in robust antitumor responses induced by PD-1 blockade (60, 106, 107).

With the advancement of systems biology methods, data derived from high-quality RNA-seq are much more convenient and cost-effective than multiplex immunohistochemistry/immunofluorescence (mIHC/IF) for interrogating a tumor transcriptome and its microenvironment. However, limitations still exist: (i) the quality of RNA extraction is easily affected by the sample storage time and temperature, resulting in high degradation, particularly when stored as FFPE tissue (108, 109); (ii) the average gene expression profile from bulk RNA-Seq may conceal the accurate signals induced by rare cell populations or cell types; and (iii) bulk RNA-seq is unable to provide spatial information regarding the TME (110).

### Single-cell RNA sequencing

Due to the complexity and heterogeneity of the TME, bioinformatics analysis based on bulk tumor gene expression profiles still cannot wholly fulfill the need for understanding the diversity of both stromal and tumor cells in the TME, particularly for undefined cell subpopulations, which prompts the emergence of single-cell technology. Single-cell RNA sequencing (scRNAseq) was first described and performed in studies by Tang et al. in 2009, in which scRNAseq on a single blastomere detected 5270 more genes than microarrays employing hundreds of blastomeres (111). In recent years, several scRNAseq methods with increasing sensitivity have been rapidly developed and intensively applied in various research fields (112, 113). Several studies have investigated the cell subclonal constitution in tumor specimens or circulation using single-cell analysis. For example, a clustering analysis of more than 5000 single cells from four responders and four nonresponders pre- and post-NAC treatment has identified CD45+EPCAM- clusters, which signify immune cell activation before NAC, as a hallmark of tumor extinction (93). Deng et al. (90) evaluated the difference in tumor-infiltrating immune cells at baseline and during treatment at the single-cell level (10x Genomics) in two metastatic TNBC patients treated with the combination of nab-paclitaxel and pembrolizumab; the enrichment of IFN+ and GZMB+ CD8+ T cells was well as TRMs was found in responders at baseline, whereas significant myeloid infiltrates accompanied by the absence of PD-1-high T cells at baseline were features of nonresponders, thereby indicating the potential prognostic value of these cell subsets as predictors for immunochemotherapy. Zhang et al. performed 10x Genomics single-cell RNA sequencing and transposase-accessible chromatin (ATAC) sequencing of tissue samples from 22 patients with advanced TNBC who had received paclitaxel chemotherapy alone or in conjunction with the anti-PD-L1 antibody atezolizumab (92); they focused on the orchestrated immune response between B cells and CXCL13-CD4 T cells, and their research results suggested a strategy of dampening antitumor CXCL13+ T cells and recruiting immunosuppressive macrophages induced by nab-paclitaxel to overcome immunochemotherapy resistance. In addition to looking for predictive cell subsets within the TME, the transcriptional changes in peripheral immune cells during NAC have also been evaluated in breast cancer patients. By performing scRNAseq (10x Genomics) on PBMCs collected during NAC, the downregulation of IFNα and IFNγ signaling pathways and the occurrence of immune exhaustion on immune cells induced by NAC have been observed (114). scRNAseq (10x Genomics) of peripheral CD8+ PD-1^hi^ T cells from TNBC patients post-NAC has identified an eight-gene score significantly associated with ongoing disease (89).

In addition to the increasing reports on how NAC remodels the TME, the evolution of malignant cells during NAC in TNBC has also been assessed utilizing scRNAseq. Using Nanogrid Single-Nucleus RNA Sequencing (WaferGen BioSystems) to analyze longitudinal samples from eight TNBC patients, Kim et al. reported that the resistant genotypes are preexisting and enriched by NAC, whereas resistance transcriptional profiles are mainly acquired during chemotherapy in TNBC patients (87). In addition, the effects of NAC on noncoding RNAs have also been explored in TNBC at the single-cell level. Shaath et al. identified the lncRNA transcriptional landscape by employing 1758 single cells from TNBC patients, and they found that the long lncRNA MALAT1 is upregulated during NAC and contributes to chemoresistance (94). With an exponential increase in the number of cells and genes, the fine resolution of single-cell transcriptomes has facilitated a deeper understanding of distinct immune subpopulation changes and cancer cell evolution under selective pressure from chemotherapy.

While scRNAseq has significantly advanced the understanding of the variability and diversity of cell subpopulations, there are still some challenges to applying scRNAseq in immuno-oncology. The accuracy is hindered by technological noise and batch effects (115). Because reanalyses of raw sequencing data in different studies are inaccessible, uploading data in public repositories should be promoted. Furthermore, due to a lack of information on posttranscriptional and posttranslational modifications, scRNAseq analysis does not always accurately reflect protein expression levels, causing conflicting evidence for the same event (116).

## Spatial transcriptomics

In addition to the transcriptional level and the constitution of cells within the TME, increasing evidence has revealed that the spatial location of cells also determines the efficacy of NAC and that NAC remodels the spatial relationship between stromal cells (117). In addition, understanding intricate spatial arrangements helps to understand how tumor cells interact, evade immune surveillance, and evolve drug resistance. The platform from 10x Genomics and the DSP from NanoString Technologies are the two commercially available spatial RNAseq technologies. A recent study has analyzed the spatial expression of 286 pharmacogenetics in 6 breast cancer tissues using the Visium spatial transcriptomics platform, which demonstrated that the expression of genes associated with reactive oxygen species (ROS) handling and detoxification mechanisms is highly heterogeneous within tumors relative to surrounding nontumor regions, particularly GPX4, GSTP1, MGST3, SOD1, CYP4Z1, CYB5R3, GSTK1, and NAT1 (118). To reveal a more comprehensive characterization of molecular diversity in mediating the chemotherapeutic response of TNBC, Kulasinghe et al. applied DSP in 24 TNBC tissue samples to quantify and analyze the differential expression of 68 targets in the tumor and TME compartments between responsive and nonresponsive tumors; elevated ER-alpha expression as well as reduced 4-1BB and MART1 expression within the stromal compartment are implicated in the adjuvant chemotherapy response, whereas increased GZMA, STING, and fibronectin levels as well as decreased CD80 levels are associated with the response within the tumor compartment (119). Similarly, DSP with a panel of 39 markers has also been applied to evaluate the expression of immune-related proteins, including MHCII, in the tumor epithelium of TNBC compared to their expression levels in the stromal compartment; higher HLA-DR levels are present in the tumor epithelial cells of patients with long-term disease-free survival and are also associated with high CD4 and ICOS levels in the stromal compartment (120).

Compared to sequencing-based techniques, such as 10x Visium and DSP, microscopic imaging-based techniques offer higher resolution but capture fewer genes. In addition, the microscopy-based approach not only achieves spatial transcript analysis of hundreds to thousands of target genes but is also compatible with immunofluorescence or DNA-coupled antibody protein reads for FFPE and fresh frozen tissues. The representative commercialization of microscopic imaging-based techniques include 10x Genomics Xenium, NanoString CosMx spatial molecular imager, and Vizgen Merscope (121, 122). Although the principles of the three technologies are based on the same *in situ* hybridization (ISH) method, each has different characteristics. The initial commercial Xenium kits achieve up to 400 gene plex, while CosMx SMI supports high plex imaging of more than 1000 RNAs (123). The Vizgen MERSCOPE Platform is the first commercial solution for MERFISH technology with imaging resolutions down to 100 nm. With the strategy of expansion-assisted iterative fluorescence ISH (EASI-FISH), the specialty of NanoString CosMX is the ability to offer a 3D resolution of gene expression in tissue and enable 50 nm subcellular resolution in the XY plane (123). These spatially resolved single-cell multiomics provide high-resolution maps of cellular subpopulations in tissue, promoting greater understanding of cell−cell interactions, cellular processes, and biomarker discovery.

The available spatial omics provide a new perspective into the spatial landscape of the tumor TME. However, the major stumbling blocks of spatial transcriptomics and proteomics technologies involve high costs, limitations due to the vast landscape of TME, and time-consuming processes (49). In addition, spatial heterogeneity, such as three types of cell distributions with heterogeneous HER2 status, cannot be disregarded (124). Thus, it is necessary to scan diverse areas, including the invasive front, core, and perimeter, which consumes more precious tissue samples. Furthermore, integrating spatial and other omics data requires developing advanced approaches with higher analytical capabilities (125). With efforts to overcome these issues, spatial omics technologies will become a vital research tool for uncovering spatially heterogeneous features that explain the resistance mechanism of NAC and the scenario of immunotherapy in breast cancer.

## Discussion

**Figure 2:**
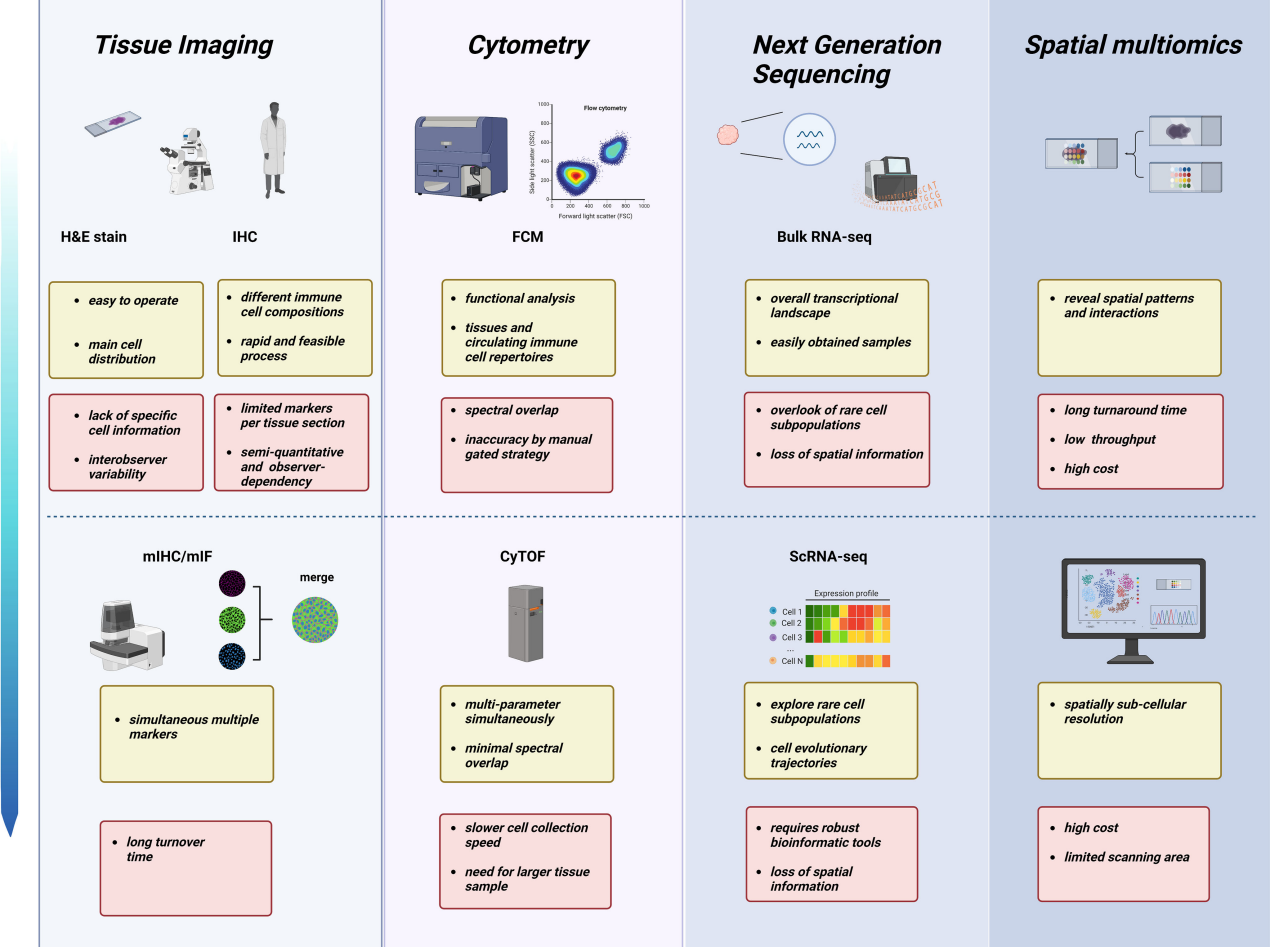
Characteristics and limitations of various technologies across different omics layers in NAC studies. From tissue pathomorphology to high-throughput sequencing, different technologies have advantages (yellow) and challenges (red). Created with BioRender.com.

Collectively, emerging high-throughput technologies and optimized bioinformatics algorithms promise the discovery of novel and more accurate biomarkers to predict the neoadjuvant therapy response and prognosis in breast cancer. In addition, series samples during NAC of TNBC also provide deep insights into how chemotherapy orchestrates immune populations in the TME and peripheral circulation, enabling the search for the dominant cell subsets or factors causing chemoresistance. However, the various techniques have advantages and disadvantages (**Figure 2**). The cost-effectiveness and practicality of these high-throughput technologies remain a major concern; additionally, analyzing large quantities of data is still time-consuming. Therefore, traditional methods, including IHC, FCM, and bulk-tumor RNA sequencing, are still predominant in clinical practice and the cancer research field. Recently, Shao et al. developed a novel IHC-based TNBC classification corresponding to the classification of TNBC based on RNA-seq (126). Multiomics analysis has also indicated that TNBC patients with higher plasma trimethylamine N-oxide (TMAO) levels achieve better responses to immunotherapy (127). These results demonstrate the robust potential of transferring multiomics research into clinical practice. Therefore, it is hopeful that prospective clinical trials will verify the key information extracted from massive high-throughput multiomics data to distinguish TNBC patients who benefit from certain treatment strategies.

## Author contributions

GW wrote the original draft of the manuscript. YY supplemented the related literature and provided significant guidance. HH and JZ critically reviewed this manuscript. CN was responsible for the conception of this review and provided funding acquisition. All authors contributed to the article and approved the submitted version.
